# Radical Cure of Hernia

**Published:** 1888-01

**Authors:** 


### RADICAL CURE OF HERNIA.

Sir William Stokes, of Dublin, reports in the *British Medical Journal*, December 3, 1887, three cases operated upon by the following method: After exposing the sac by the usual herniotomy incision, and dissecting it away from the spermatic cord, it is ligated in two places and divided between. The sac is then seized with forceps and twisted until resistance is felt, as recommended by C. B. Ball.

Two silk sutures are then introduced, each passing through the pillars and walls of the canal, nearer the inner than the external ring, and each transfixing the twisted sac.

These sutures are brought through the skin one inch from the margins of the wound and tied in button fashion. The skin is united by superficial suture.

Mr. Kendal Franks, of Dublin, recommends an incision on a higher plane, but in the same direction, than the inguinal canal, and closure of the internal and external rings by buried sutures of silver wire. Those for the internal ring are passed through the external oblique, Poupart's ligament, the internal oblique and the sac, and out through the conjoined tendon and external oblique on the opposite side of the canal. Two wires are used. A third wire closes the external ring.

Forty-one operations for radical cure of hernia are reported by Mr. A. E. Baker. The operator uses silk for suture in preference to catgut or kangaroo tendon. His method is to ligate and divide the sac, leaving the scrotal portion in position, then to invaginate the ligatured stump and suture the pillars. A case of strangulated femoral hernia containing an ovary and fallopian tube, operated upon with removal of these structures and suture of the sac, is reported to have been followed by complete recovery.

